# Notes upon New Remedies

**Published:** 1887-07

**Authors:** 


### NOTES UPON NEW REMEDIES.—WIESBADEN.

Wiesbaden medicinal springs have been famous for centuries as a resort for the afflicted, they being considered most efficacious in gout, rheumatism, and skin diseases. The water contains numerous salts, the chief being the chloride of sodium; next in amount are the chloride of calcium, and the carbonate of lime. To those who believe in the efficacy of "tissue remedies" this water offers a compound of many of them with carbonates of copper, barium, strontium, and iron thrown in.

Imperfect provings of this water have been arranged for us in the *Encyclopædia.* From this account we gather that this remedy has no very distinctive symptoms. New remedies are of little value unless they present something which the old and better proven ones do not afford.

Under the mental symptoms we find nothing worthy of note; a marked vertigo was cured by the baths. The water has a strong effect upon the scalp and the skin; it seems to promote desquamation and to aid in forming a healthy skin. We find the hair of the scalp falls out rapidly and the new growth is rapid and thick (resembling Cepa, Lyc., Mezer.). Eyebrows and eyelashes are similarly affected. The skin desquamates rapidly and becomes soft and healthy. Corns become soft and fall off! an effect, we believe, not credited to any other water. This effect followed most cases from continuous bathing; provings should be made with potencies to ascertain the true sphere of the remedy. The ears and nose both itch and both have increased secretions. The tongue is coated, chiefly a brown color, which, in one case, disappeared after drinking coffee; we find that coffee also increased the secretion of urine.

Under stomach, appetite, nausea, etc., we find nothing of special value. Stool and flatus are apt to be offensive. The stool is generally thin, slimy, of dark color and offensive; is worse after drinking (resembling in this *Aloe, Arg-n., Ars., Crot-tig., Thromb.*, etc.). It also resembles Aloe in being involuntary when urinating or emitting flatus, also by occurring early in morning. The Aloe patient is troubled by the fear that the stool will escape at any time; it seems to be *very* insecurely retained.

The urine, as before noted, is more copious after coffee; another peculiarity is that it "makes the linen stiff like starch." The menses are increased and become earlier; a woman liable to miscarriage seems to have been benefited; another hitherto sterile became pregnant for first time after its use.

Under respiratory organs we notice: expectoration always occurs after drinking the water, very seldom at other times; oppression of chest and of breathing whenever chest was covered in the bath, obliged bather to raise chest out of water. Constriction of chest from the (loose) clothes. (Apis, Bell., Lach., etc., have this oppressed feeling from seemingly tight neck-wear.)

The nails grow rapidly (also *Fluor-ac.* and Kali iod.). Corns become softer, fall off, or are readily removed.

The strength is increased; is able to walk for hours without fatigue (here again resembling Fluor-ac. and Kali iod., both of these remedies having great desire to walk in open air, and ability to do so without fatigue).

Besides the free desquamation and subsequent improved condition of the skin, we find great itching followed bathing. The baths produce eruptions of the simpler forms, perspiration which seems to relieve the itching.

